# The effect of a pharmaceutical ghrelin agonist on lifespan in C57BL/6J male mice: A controlled experiment

**DOI:** 10.1111/acel.13787

**Published:** 2023-02-03

**Authors:** Kathryn A. Kaiser, Inga Kadish, Thomas van Groen, Daniel L. Smith, Stephanie Dickinson, Beate Henschel, Erik S. Parker, Andrew W. Brown, David B. Allison

**Affiliations:** ^1^ Department of Health Behavior, School of Public Health University of Alabama at Birmingham Birmingham Alabama USA; ^2^ Department of Cell, Developmental and Integrative Biology, School of Medicine University of Alabama at Birmingham Birmingham Alabama USA; ^3^ Department of Nutrition Sciences, School of Health Professions University of Alabama at Birmingham Birmingham Alabama USA; ^4^ Department of Epidemiology and Biostatistics, School of Public Health Indiana University‐Bloomington Bloomington Indiana USA; ^5^ Department of Biostatistics University of Arkansas for Medical Sciences Little Rock Arkansas USA

**Keywords:** aging, ghrelin agonist, lifespan, mice

## Abstract

Interventions for animal lifespan extension like caloric restriction (CR) have identified physiologic and biochemical pathways related to hunger and energy‐sensing status as possible contributors, but mechanisms have not been fully elucidated. Prior studies using ghrelin agonists show greater food intake but no effect on lifespan in rodent models. This experiment in male C57BL/6J mice tested the influence of ghrelin agonism for perceived hunger, in the absence of CR, on longevity. Mice aged 4 weeks were allowed to acclimate for 2 weeks prior to being assigned (*N* = 60/group). Prior to lights off daily (12:12 cycle), animals were fed a ghrelin agonist pill (LY444711; Eli Lilly) or a placebo control (Ctrl) until death. Treatment (GhrAg) animals were pair‐fed daily based on the group mean food intake consumed by Ctrl (ad libitum feeding) the prior week. Results indicate an increased lifespan effect (log‐rank *p* = 0.0032) for GhrAg versus placebo Ctrl, which weighed significantly more than GhrAg (adjusted for baseline weight). Further studies are needed to determine the full scope of effects of this ghrelin agonist, either directly via increased ghrelin receptor signaling or indirectly via other hypothalamic, systemic, or tissue‐specific mechanisms.

## Graphical abstract

In this experiment, C57BL/6J mice (*N* = 60/group, males) were tested for the influence of ghrelin agonism for perceived hunger, without caloric restriction, on longevity. Mice were fed a ghrelin agonist (LY444711; Eli Lilly) or placebo (Ctrl) until death. Treatment (GhrAg) animals were pair‐fed daily based on the group mean food intake consumed by Ctrls (ad libitum feeding) the prior week. Results indicate an increased lifespan effect (log‐rank *p* = 0.0032) for GhrAg mice. Body weight differences may contribute to the outcomes.

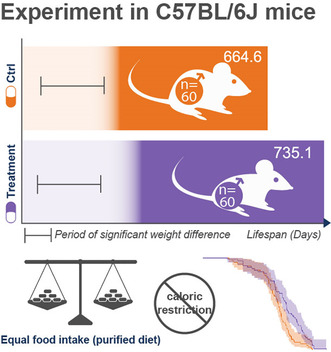

**Abbreviations**
BW: body weight
CR: caloric restriction
GhrAg: ghrelin agonist
GHS‐R: growth hormone secretagogue receptor

## 1 INTRODUCTION, RESULTS AND DISCUSSION

Of the well‐studied effects on lifespan in mouse models, detailed mechanisms underlying the health and longevity benefit of caloric restriction (CR) are still being investigated despite some limitations on the practical applications to humans (Longo et al., 2015; Sharples et al., 2015). A major limitation of animal models is that they cannot self‐report hunger or other physiological sensations that would inform mechanistic work. Since ghrelin was first described and noted as a growth hormone secretagogue receptor (GHS‐R) agonist (Kojima et al., 1999), much study has focused on the effects on hunger and appetite regulation along with other aspects of energy balance (Bouillon‐Minois et al., 2021; Lewiński et al., 2021; Ouerghi et al., 2021).

Investigators examining effects on cognition in mouse models have posited that the mechanism may involve interoceptive cues or signaling, rather than reduced energy intake per se (Dhurandhar et al., 2013; Kunath et al., 2015). In those studies, oral administration of a ghrelin agonist (LY444711, an orally active compound that binds with high affinity to and is a potent activator of the growth hormone secretagogue receptor 1a [GHS‐R1a] receptor; Lugar et al., 2004), reduces Alzheimer's disease pathology and improves cognition in the APP‐SwDI mouse model (Dhurandhar et al., 2013). Treatment also reduced levels of amyloid beta (Aβ) and neuroinflammation (as measured by microglial activation) at 6 months of age compared to controls (Ctrls), like the effect seen in the 20% CR group (gp) but with no significant difference in body weight (BW) or % body fat (Dhurandhar et al., 2013).

LY444711 binds to the human ghrelin receptor (GHS‐R1a) and is a functional agonist. This agonist (Lugar et al., 2004) produced orexigenic behavior in rodents, including stimulated energy intake (food consumption; 40%> than Ctrl at 10 mg/kg, 50%> than Ctrl at 30 mg/kg dose), positive energy balance (23%> BW with 2 weeks treatment at 10 mg/kg), acute higher respiratory quotient (RQ) with increased dose (3, 10, and 30 mg/kg) and increased adiposity (greater fat mass but no significant difference in lean mass by DXA; Tschöp et al., 2000). The authors conclude this substance is orally active with an extended half‐life relative to native ghrelin. Despite various studies on ghrelin effects on food intake and body composition, studies of ghrelin agonists on lifespan‐extending effects in rodent models are lacking. We tested the hypothesis that a pathway related to perceived hunger, as induced by an oral, exogenously administered orexigenic agent (i.e., a synthetic ghrelin agonist), would differentially affect lifespan in mice when pair‐fed to Ctrls.

## 2 RESULTS

### 2.1 Food intake

**FIGURE 1:**
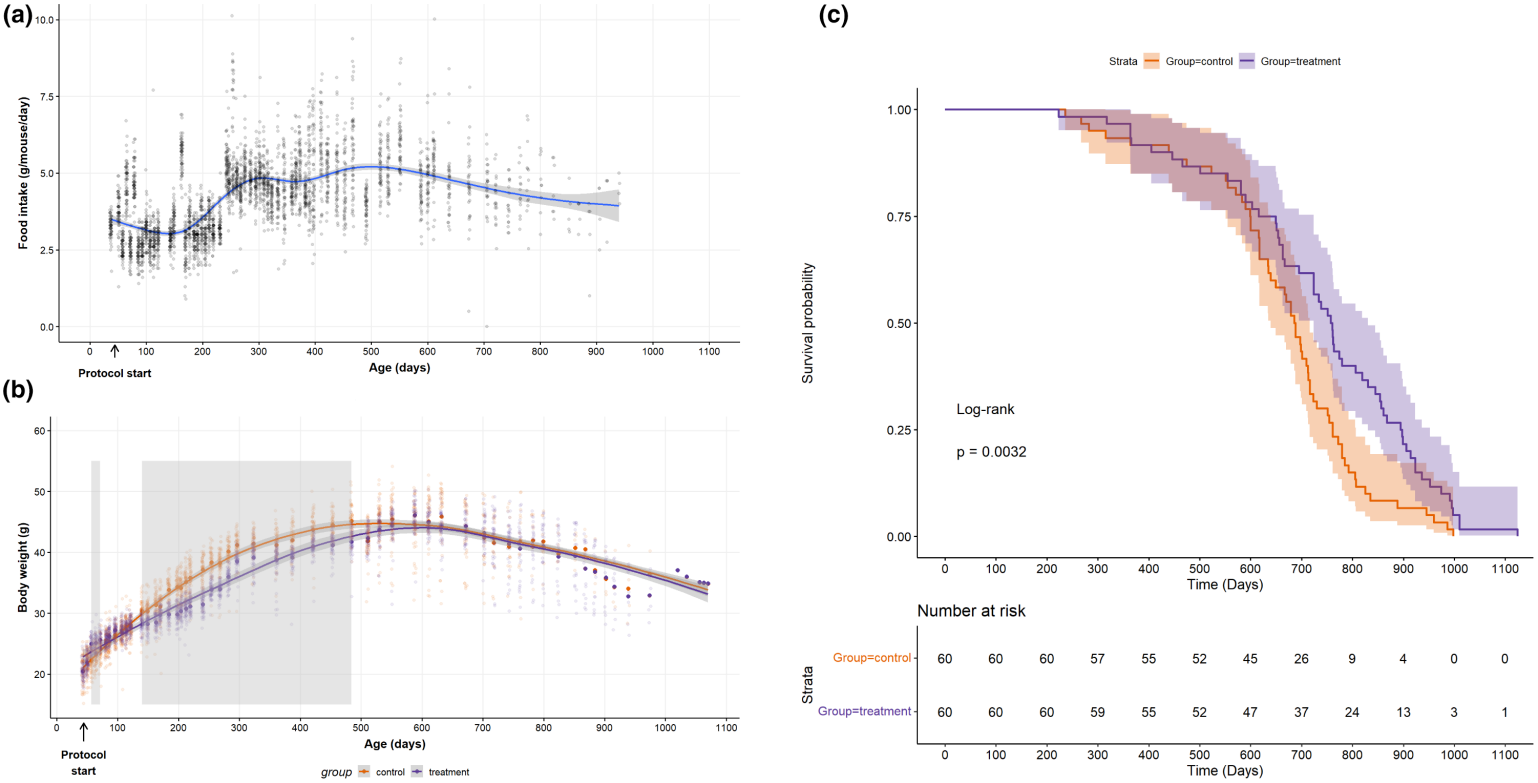
(a) Mean food intake (g/mouse/day) of the Ctrl gp; (b) BW by gp (g) over the duration of the protocol; (c) lifespan outcomes by gp. For the first 88 days, the GhrAg pill concentration was 1.66% (by weight), after which, it was increased to 2.4% to achieve the 30 mg/kg dose. Diet was initially AIN‐93G for ~6 months, then switched to AIN‐93M for the remainder of the experiment. *Note*: Shaded vertical areas in (b) indicate periods where gp means were significantly different for greater than one measurement interval. Some individual measurements that were significantly different but noncontiguous are not shaded.

Food intake before the start of the protocol was significantly greater in Ctrls (3.25 ± 0.35 g/day) compared to GhrAg animals (2.94 ± 0.61 g/day, *p* = 0.001, 95% CI for the difference = 0.13–0.49 g). On protocol, per study design, the Ctrl gp mean food intake was pair‐fed to the GhrAg gp – Ctrl intake in Figure 1a.

### 2.2 Body weight

Despite food intake differences, baseline BW did not statistically differ (Ctrl: 20.71 ± 1.02 g, GhrAg: 20.37 ± 1.01 g, *p* = 0.07, CI: −0.02 to 0.71 g). At times, mean gp BW diverged and converged (Figure 1b). Gp × time differences were observed when adjusting for baseline BW of individual animals [*F*(51, 4489) = 18.3, *p* < 0.001]. Age at maximum BW was not significantly different between the two gps (Ctrl: 483 ± 136 days, GhrAg: 494 ± 131 days, *p* = 0.648).

### 2.3 Longevity

Overall, GhrAg animals lived significantly longer than Ctrls (log‐rank test *p* = 0.003, unadjusted Cox proportional hazards model *p* = 0.004, Figure 1c), which remained significant after adjusting for baseline BW (*p* = 0.012). However, the details over the lifespans are complex. The age when the GhrAg animals achieve both the 50th and 90th quantiles of survival is later than the Ctrl animals (Ctrl: 688 [50th] and 835 days [90th], GhrAg: 757 [50th] and 974 days [90th]), while the difference between the gps in quantile regression is statistically significant at the 90th quantile (*p* = 0.023 at 90th quantile) but not the 50th (*p* = 0.069). Maximum lifespan analysis via Gao‐Allison at the 90th percentile did not show a significant gp difference (*p* = 0.199). In general linear models on longevity, differences by gp were significant (*p* = 0.035): the GhrAg‐treated gp had longer lifespans compared to the Ctrls. For all animals regardless of gp, later age at max BW was predictive of longevity: for each 1 day later that max BW was reached, lifespan was extended by 0.95 day [*F*(1, 118) = 74.87, *p* < 0.001]. There was no significant gp main effect (*p* = 0.657) or gp by age at maximum BW interaction (*p* = 0.225).

### 2.4 Observed pathologies

We saw gp differences in those requiring euthanasia per veterinary protocols (Table 1): *χ* ^2^ (1) = 4.48, *p* = 0.034. The largest difference in pathologies observed in gross necropsy was the higher rate of enlarged seminal vesicles in the GhrAg gp, and they also exhibited higher rates of being moribund, resulting in euthanasia. The most common health issue observed in both gps was ulcerative dermatitis, some of which was resolved after treatment. See Supplement for additional details of how animal health status was monitored.

**TABLE 1:** Counts of death conditions and rates of ulcerative dermatitis and gross necropsy observations by gp.

Gp death condition	Ctrl (*n* = 60)	GhrAg (*n* = 60)
Found dead^a^	26	15
Euthanized	34	45
Gross necropsy observations		
Ulcerative dermatitis	29	27
Liver	17	18
Spleen	5	6
Seminal vesicles^b^	2	13
Moribund^b^	2	11
Other^c^	11	13

^a^ Between gps found dead versus euthanized *χ* ^2^ (1) = 4.48, *p* = 0.034.

^b^ Not tested due to fewer than 5 cases per cell.

^c^ Other observations of the euthanized animals included blood pooling, tumors, and abdominal and bladder abnormalities.

## 3 DISCUSSION

The results of this experiment indicate the ghrelin agonist with weekly adjusted pair‐feeding lengthened lifespan and decreased BW. Since the GhrAg gp were pair‐fed to Ctrl gp's mean ad libitum (AL) food intake, it is not known whether GhrAg animals perceived hunger or could be considered to be under a state of negative energy balance as reflected in lower BW at times compared to Ctrls. In another study, LY444711 treatment for 2 weeks increased adiposity by stimulating food consumption and limiting fat utilization in male rats, while increasing RQ and carbohydrate utilization during the dark phase (Lugar et al., 2004). This possible ‘perception’ of inadequate food resources, even at low levels, may stimulate adaptive mechanisms to change body composition, which may also affect rates of aging (Kaiser et al., 2012). All GhrAg‐treated animals consumed all food delivered daily in the present study. This amount was, at times, higher than observed in many other studies of the same sex of this mouse strain but may be attributed to the single‐housing condition (Rowland et al., 2018; Schipper et al., 2018). It is unknown whether GhrAg animals would show a similar degree of hyperphagia over the lifespan as has been observed in acute studies.

Strengths of this study include a rigorous experimental design, including the single chemical identity tested, dosing related to similar diurnal patterns in humans, diet provision and monitoring with energy intake matched gps, strain historic context and information for a normative longevity profile and large sample size. Limitations of interpretation include the use of an inbred strain, use of only male mice, AL feeding variability among Ctrls with the treatment animals all being pair‐fed to the Ctrl gp mean, time of food access (24 h in AL), feeding behaviors (daily provision with agonist), no ability to make comparisons with the time of day feeding, and long duration of GhrAg exposure. We did not systematically compare health factors to quantify the health span between groups. Increased physical activity has been observed in short‐term experiments with this same ghrelin agonist but was not measured over the entire protocol here. Future studies may wish to consider machine‐assisted analysis of movement to enhance the prediction of longevity outcomes, as reported by Hession et al. (2022).

### 3.1 Other ghrelin‐related GHS‐R agonists show similar effects in rats

Similar to the Lilly compound (LY444711), high‐dose treatment with single agonist compounds (BIM‐28131 and BIM‐28125, which have higher binding affinity to ghrelin receptor GHS‐1a than natural ghrelin) or exogenous human ghrelin have been tested in rat models, resulting in increased BW gain by promoting fat mass, with only BIM‐28131 showing a significantly increased lean mass (Strassburg et al., 2008). Food intake increased during treatment with BIM‐28131 or ghrelin, but no effects on BW–adjusted energy expenditure were observed over the 4‐week study. With the lower dose, only BIM‐28131 had a significant effect on BW. These results characterize BIM‐28131 as a promising GHS‐R agonist with an attractive action profile for the GhrAg of catabolic disease states such as cachexia (Strassburg et al., 2008).

Reports of mechanistic studies show increased hypothalamic SIRT1 activity and SIRT1 protein expression in heart tissue when mice models were exposed to ghrelin signaling potentiators such as rikkunshito and atractylodin (Fujitsuka et al., 2016). Recent evidence in *Drosophila melanogaster* shows interesting linkages between CR and tissue‐specific aging (CLOCK gene regulation in eyes) that extend lifespan (Hodge et al., 2022). Work in *Caenorhabditis elegans* demonstrates the roles of serotonin and dopamine receptor antagonism in mimicking CR that result in increased longevity by way of the Fmo genes (Miller et al., 2022). Mammals and *C. elegans* share a common ancestral Fmo gene (Petalcorin et al., 2005).

## 4 CONCLUSIONS

These observations suggest ghrelin as a growth hormone secretagogue may influence a network of factors related to aging or body composition. Greater focus on tissue‐specific effects, neurotransmitters, gene expression (e.g., UCP2; Wang et al., 2010), and metabolic phenotypes (e.g., body temperature, physical activity, diet composition effects and potential sex differences) as well as use of composite frailty index measures (Kane et al., 2016) are warranted.

## AUTHOR CONTRIBUTIONS

Kathryn A. Kaiser – Data curation, Formal analysis, Project administration, Validation, Visualization, Writing – original draft, Writing – review & editing. Inga Kadish – Conceptualization, Data curation, Investigation, Methodology, Project administration, Resources, Supervision, Writing – review & editing. Thomas van Groen – Conceptualization, Data curation, Investigation, Methodology, Project administration, Resources, Supervision. Daniel L. Smith, Jr. – Conceptualization, Formal analysis, Investigation, Methodology, Project administration, Writing – original draft, Writing – review & editing. Stephanie Dickinson – Data curation, Formal analysis, Validation, Visualization, Writing – review & editing. Beate Henschel – Data curation, Formal analysis, Validation, Visualization, Writing – review & editing. Erik S. Parker – Data curation, Formal analysis, Validation, Visualization, Writing – review & editing. Andrew W. Brown – Formal analysis, Writing – review & editing. David B. Allison – Conceptualization, Formal analysis, Funding acquisition, Project administration, Resources, Writing – review & editing.

## ACKNOWLEDGEMENTS

The authors are grateful to Dr. Steve Austad for comments on an earlier draft of this manuscript.

## FUNDING INFORMATION

Research reported in this publication was supported by the National Institute on Aging of the National Institutes of Health under awards R01AG043972, P30DK056336, P30 AG050886, and P30NS47466. The content is solely the responsibility of the authors and does not necessarily represent the official views of the National Institutes of Health.

## CONFLICT OF INTEREST STATEMENT

None of the authors have any relevant items to disclose related to this research. The opinions expressed are those of the authors and do not necessarily represent those of the NIH or any other organization. The ghrelin agonist used was donated by Eli Lilly and Company to UAB. Indiana University has received grants, contracts, and in‐kind donations from Eli Lilly and Company for additional scientific and educational projects.

### OPEN RESEARCH BADGES

This article has earned an Open Data badge for making publicly available the digitally‐shareable data necessary to reproduce the reported results. The data is available at [https://osf.io/34cr8/].

## Supporting information


Appendix S1

## Data Availability

Data and analytic code for the project entitled ‘Aim 5 – Ghrelin agonist longevity outcome’ are available here: https://osf.io/34cr8/. Data are available under the terms of CC BY‐NC‐SA 4.0 by the authors.

## References

[acel13787-bib-0001] Bouillon‐Minois, J. B. , Trousselard, M. , Thivel, D. , Gordon, B. A. , Schmidt, J. , Moustafa, F. , Oris, C. , & Dutheil, F. (2021). Ghrelin as a biomarker of stress: A systematic review and meta‐analysis. Nutrients, 13(3), 1–15. 10.3390/nu13030784 PMC7997253 33673594

[acel13787-bib-0002] Dhurandhar, E. J. , Allison, D. B. , van Groen, T. , & Kadish, I. (2013). Hunger in the absence of caloric restriction improves cognition and attenuates Alzheimer's disease pathology in a mouse model. PLoS One, 8(4), e60437. 10.1371/journal.pone.0060437 23565247 PMC3614512

[acel13787-bib-0003] Fujitsuka, N. , Asakawa, A. , Morinaga, A. , Amitani, M. S. , Amitani, H. , Katsuura, G. , Sawada, Y. , Sudo, Y. , Uezono, Y. , Mochiki, E. , Sakata, I. , Sakai, T. , Hanazaki, K. , Yada, T. , Yakabi, K. , Sakuma, E. , Ueki, T. , Niijima, A. , Nakagawa, K. , … Inui, A. (2016). Increased ghrelin signaling prolongs survival in mouse models of human aging through activation of sirtuin1. Molecular Psychiatry, 21(11), 1613–1623. 10.1038/mp.2015.220 26830139 PMC5078860

[acel13787-bib-0004] Hession, L. E. , Sabnis, G. S. , Churchill, G. A. , & Kumar, V. (2022). A machine‐vision‐based frailty index for mice. Nature Aging, 2(8), 756–766. 10.1038/s43587-022-00266-0 PMC10117690 37091193

[acel13787-bib-0005] Hodge, B. A. , Meyerhof, G. T. , Katewa, S. D. , Lian, T. , Lau, C. , Bar, S. , Leung, N. Y. , Li, M. , Li‐Kroeger, D. , Melov, S. , Schilling, B. , Montell, C. , & Kapahi, P. (2022). Dietary restriction and the transcription factor clock delay eye aging to extend lifespan in *Drosophila melanogaster* . Nature Communications, 13(1), 3156. 10.1038/s41467-022-30975-4 PMC9174495 35672419

[acel13787-bib-0006] Kaiser, K. A. , Smith, D. L., Jr. , & Allison, D. B. (2012). Conjectures on some curious connections among social status, calorie restriction, hunger, fatness, and longevity. Annals of the new York Academy of Sciences, 1264(1), 1–12. 10.1111/j.1749-6632.2012.06672.x 22834696 PMC3464393

[acel13787-bib-0007] Kane, A. E. , Hilmer, S. N. , Boyer, D. , Gavin, K. , Nines, D. , Howlett, S. E. , de Cabo, R. , & Mitchell, S. J. (2016). Impact of longevity interventions on a validated mouse clinical frailty index. The Journals of Gerontology. Series A, Biological Sciences and Medical Sciences, 71(3), 333–339. 10.1093/gerona/glu315 25711530 PMC4757961

[acel13787-bib-0008] Kojima, M. , Hosoda, H. , Date, Y. , Nakazato, M. , Matsuo, H. , & Kangawa, K. (1999). Ghrelin is a growth‐hormone‐releasing acylated peptide from stomach. Nature, 402(6762), 656–660. 10.1038/45230 10604470

[acel13787-bib-0009] Kunath, N. , van Groen, T. , Allison, D. B. , Kumar, A. , Dozier‐Sharpe, M. , & Kadish, I. (2015). Ghrelin agonist does not foster insulin resistance but improves cognition in an Alzheimer's disease mouse model. Scientific Reports, 5(1), 11452. 10.1038/srep11452 26090621 PMC4473679

[acel13787-bib-0010] Lewiński, A. , Karbownik‐Lewińska, M. , Wieczorek‐Szukała, K. , Stasiak, M. , & Stawerska, R. (2021). Contribution of ghrelin to the pathogenesis of growth hormone deficiency. International Journal of Molecular Sciences, 22(16), 1–21. 10.3390/ijms22169066 PMC8396656 34445772

[acel13787-bib-0011] Longo, V. D. , Antebi, A. , Bartke, A. , Barzilai, N. , Brown‐Borg, H. M. , Caruso, C. , Curiel, T. J. , de Cabo, R. , Franceschi, C. , Gems, D. , Ingram, D. K. , Johnson, T. E. , Kennedy, B. K. , Kenyon, C. , Klein, S. , Kopchick, J. J. , Lepperdinger, G. , Madeo, F. , Mirisola, M. G. , … Fontana, L. (2015). Interventions to slow aging in humans: Are we ready? Aging Cell, 14(4), 497–510. 10.1111/acel.12338 25902704 PMC4531065

[acel13787-bib-0012] Lugar, C. W. , Clay, M. P. , Lindstrom, T. D. , Woodson, A. L. , Smiley, D. , Heiman, M. L. , & Dodge, J. A. (2004). Synthesis and biological evaluation of an orally active ghrelin agonist that stimulates food consumption and adiposity in rats. Bioorganic & Medicinal Chemistry Letters, 14(23), 5873–5876. 10.1016/j.bmcl.2004.09.027 15501059

[acel13787-bib-0013] Miller, H. A. , Huang, S. , Dean, E. S. , Schaller, M. L. , Tuckowski, A. M. , Munneke, A. S. , Beydoun, S. , Pletcher, S. D. , & Leiser, S. F. (2022). Serotonin and dopamine modulate aging in response to food odor and availability. Nature Communications, 13(1), 3271. 10.1038/s41467-022-30869-5 PMC9174215 35672307

[acel13787-bib-0014] Ouerghi, N. , Feki, M. , Bragazzi, N. L. , Knechtle, B. , Hill, L. , Nikolaidis, P. T. , & Bouassida, A. (2021). Ghrelin response to acute and chronic exercise: Insights and implications from a systematic review of the literature. Sports Medicine, 51(11), 2389–2410. 10.1007/s40279-021-01518-6 34374968 PMC8514378

[acel13787-bib-0015] Petalcorin, M. I. , Joshua, G. W. , Agapow, P. M. , & Dolphin, C. T. (2005). The fmo genes of Caenorhabditis elegans and C. briggsae: Characterisation, gene expression and comparative genomic analysis. Gene, 346, 83–96. 10.1016/j.gene.2004.09.021 15716098

[acel13787-bib-0016] Rowland, N. E. , Robertson, K. L. , Minaya, D. , Minervini, V. , Cervantez, M. , Kaiser, K. A. , & Allison, D. B. (2018). Effect of food predictability on life span in male mice. The Journals of Gerontology: Series A, 74(8), 1158–1161. 10.1093/gerona/gly231 PMC6625587 30289438

[acel13787-bib-0017] Schipper, L. , Harvey, L. , van der Beek, E. M. , & van Dijk, G. (2018). Home alone: A systematic review and meta‐analysis on the effects of individual housing on body weight, food intake and visceral fat mass in rodents. Obesity Reviews, 19(5), 614–637. 10.1111/obr.12663 29334694

[acel13787-bib-0018] Sharples, A. P. , Hughes, D. C. , Deane, C. S. , Saini, A. , Selman, C. , & Stewart, C. E. (2015). Longevity and skeletal muscle mass: The role of IGF signalling, the sirtuins, dietary restriction and protein intake. Aging Cell, 14(4), 511–523. 10.1111/acel.12342 25866088 PMC4531066

[acel13787-bib-0019] Strassburg, S. , Anker, S. D. , Castaneda, T. R. , Burget, L. , Perez‐Tilve, D. , Pfluger, P. T. , Nogueiras, R. , Halem, H. , Dong, J. Z. , Culler, M. D. , Datta, R. , & Tschop, M. H. (2008). Long‐term effects of ghrelin and ghrelin receptor agonists on energy balance in rats. American Journal of Physiology. Endocrinology and Metabolism, 295(1), E78–E84. 10.1152/ajpendo.00040.2008 18460598 PMC2493589

[acel13787-bib-0020] Tschöp, M. , Smiley, D. L. , & Heiman, M. L. (2000). Ghrelin induces adiposity in rodents. Nature, 407(6806), 908–913. 10.1038/35038090 11057670

[acel13787-bib-0021] Wang, Y. , Nishi, M. , Doi, A. , Shono, T. , Furukawa, Y. , Shimada, T. , Furuta, H. , Sasaki, H. , & Nanjo, K. (2010). Ghrelin inhibits insulin secretion through the AMPK–UCP2 pathway in β cells. FEBS Letters, 584(8), 1503–1508. 10.1016/j.febslet.2010.02.069 20206170

