# Supplementary material for: The effect of a pharmaceutical ghrelin agonist on lifespan in C57BL/6J male mice: A controlled experiment
Source: Aging Cell. 2023 Feb 3;22(4):e13787. doi: 10.1111/acel.13787 (PMC10086516; doi:10.1111/acel.13787)
Supplement: Supplementary file 1 — Appendix S1 [file ACEL-22-e13787-s001.docx]

The effect of a pharmaceutical ghrelin agonist on lifespan in C57BL/6J male mice: A controlled experiment

Supplemental Information

Kathryn A. Kaiser, PhD, Inga Kadish, PhD, Thomas van Groen, PhD, Daniel L. Smith, Jr., PhD, Stephanie Dickinson, MS, Beate Henschel, MPH, Erik S. Parker, PhD, Andrew W. Brown, PhD, David B. Allison, PhD

*Methods*

120 C57BL/6J male mice aged 6 weeks (acquired at 4 weeks, acclimated for 2 weeks) were allocated by weight matching in a 1:1 ratio (60/group, longevity was the outcome used to determine sample size, single animals were the unit of analysis) to receive either a 30 mg/kg of synthetic ghrelin (LY444711, provided at no cost by Eli Lilly and Company, Indianapolis, IN) incorporated into a 45 mg sucrose pill (GhrAg) or a sucrose pill without a ghrelin agonist (Ctrl). All animals received the treatment or control pill daily just prior to lights out. Food was given once the pill consumption was verified. For the first 88 days of the protocol, the GhrAg pill concentration was 1.66% (by weight), after which, it was increased to 2.4% to achieve the 30 mg/kg dose. All animals were singly housed in the same room in the animal care facility at the University of Alabama at Birmingham (UAB). The protocol was reviewed and approved by the UAB IACUC.

Animals were kept on a 12-h light:12-h dark cycle (lights on at 6AM) for 2 weeks to acclimate to conditions prior to initiation of the experimental protocol. Following acclimation, mice were allocated into two groups based on weight matching by IK. The Ctrl group receive *ad libitum* access to food while the GhrAg group received the same mean amount of diet consumed by the Ctrl group in the prior week as daily provisions. Food intake was measured at weekly intervals in the Ctrl group during the first year (monthly thereafter) and based on the calculated mean daily intake, GhrAg group animals were pair fed accordingly. The purified diet for both groups was AIN-93G [(Reeves et al., 1993) growth formula, TestDiet, St. Louis, MO, USA] for 180 days, and then it was switched to AIN-93M (maintenance formula) for the remainder of the experimental protocol in keeping with recommendations as given in (Reeves, 1997). In the development of the diets by the Animal Institute of Nutrition, these diets were formulated for the different life stages of rodents, which has relevance in the context of long-term study designs as we report here. AIN 93-G has 16.6 kjoule/g (kJ/g) of metabolizable energy (typical analysis) and 16.3 kJ/g (calculated based on individual ingredients). AIN-93M has 15.8 kJ/g of metabolizable energy (typical analysis) and 16.3 kJ/g (calculated based on individual ingredients).

All animals were housed in a single, dedicated room. Water was freely available, all cages had standard bedding with cardboard nesting tubes; later, these were transitioned to Nestlets (Lab Supply, Fort Worth, TX, USA) for both groups. Body weight was measured in all animals 2–3 times monthly during the first year of life and then monthly, thereafter. Animals lived out their natural lifespans or were euthanized as necessary when health issues were observed, according to the UAB IACUC protocols and guidelines. These guidelines include requiring veterinary attention when certain postural, locomotor and body condition scoring observations are made. Additionally, ulcerative dermatitis, hair loss, rectal prolapse and presence of tumors require veterinary examination and treatment plans. Animals deemed to have body condition scores at or below 2 are euthanized. Animals showing signs of dermatitis were treated with topical agents and nail trimming, as appropriate, until resolution. Gross necropsies were performed on all animals, whether found dead or euthanized, and major observations were recorded. Other work on other longevity promoting interventions have not shown these to result in statistically different outcomes compared to controls other than those related to body weight, temperature, fur-related states, eyes and hearing (Kane et al., 2016). Thus, we limited our assessments of health status to those recommended by the UAB IACUC.

*Statistical Analysis*

All animals’ data were analyzed using R software (R Core Team, 2020). The lifespans of the two groups (GhrAg and Ctrl) were compared using Kaplan-Meier curves with non-parametric log-rank test, Cox proportional hazard regression models, maximum lifespan analysis, and quantile regression. Primary survival analyses were performed for all-cause mortality (intent-to-treat model with no censoring). The proportional hazards assumption was tested and satisfied (*p*>0.05) for the Cox models, for group, and additionally adjusted for body weight at baseline (Grambsch & Therneau, 1994). Maximum lifespan based on the 90^th^ percentile of survival was performed (Gao et al., 2008).

The Gao-Allison test uses the Wilcoxon-Mann-Whitney test to compare values for variable Z between groups of all animals where Z equals the observed lifespan if lifespan is above the 90^th^ percentile and 0 otherwise. Additionally, quantile regressions at the 50^th^ and 90^th^ quantiles were performed. The study was designed to last until all the animals died, permitting analyses by general linear models to compare the overall lifespans between groups, with age at maximum body weight and group by age at maximum weight interaction as predictors.

Energy intake, body weight at baseline, and age at maximum body weight were compared between control and treated animals with Welch two-sample t-tests. Body weight between the two groups was analyzed with linear mixed models with treatment group, baseline body weight, day, and group-by-day interaction as covariates. To account for repeated measurements of body weight, random effects for animal were included in the model with an unstructured covariance structure. Gross necropsy observations were tabulated and compared with a χ^2^ test. Unless otherwise noted, results are presented as mean ± standard deviation.

Model assumptions of normality and equal variance of residuals were evaluated for lifespan and body weight analyses and were considered satisfied with skewness <2 and ratio of variances <2 as well as balanced sample sizes between groups (Glass & Hopkins, 1996; Glass et al., 1972). A sensitivity analysis excluding observations with studentized residuals >3 and <-3 was conducted to assess the impact of outliers in the body weight analyses. Results were similar with the same significance levels observed (data not shown).

**Bibliography**

Gao, G., Wan, W., Zhang, S., Redden, D. T., & Allison, D. B. (2008). Testing for differences in distribution tails to test for differences in 'maximum' lifespan. *BMC Med Res Methodol*, *8*, 49. <https://doi.org/10.1186/1471-2288-8-49>

Glass, G., & Hopkins, K. D. (1996). *Statistical methods in psychology and education* (3rd ed.). Allyn & Bacon.

Glass, G. V., Peckham, P. D., & Sanders, J. R. (1972). Consequences of Failure to Meet Assumptions Underlying the Fixed Effects Analyses of Variance and Covariance. *Review of Educational Research*, *42*, 237 - 288.

Grambsch, P., & Therneau, T. (1994). Proportional hazards tests and diagnostics based on weighted residuals. *Biometrika*, *81*, 515-526.

Kane, A. E., Hilmer, S. N., Boyer, D., Gavin, K., Nines, D., Howlett, S. E., de Cabo, R., & Mitchell, S. J. (2016). Impact of Longevity Interventions on a Validated Mouse Clinical Frailty Index. *J Gerontol A Biol Sci Med Sci*, *71*(3), 333-339. <https://doi.org/10.1093/gerona/glu315>

R Core Team. (2020). *R: A language and environment for statistical computing.* . In R Foundation for Statistical Computing, Vienna, Austria. <https://www.R-project.org/>

Reeves, P. G. (1997). Components of the AIN-93 Diets as Improvements in the AIN-76A Diet. *Journal of Nutrition*, *127*(5), 838S-841S.

Reeves, P. G., Nielsen, F. H., & Fahey, G. C., Jr. (1993). AIN-93 purified diets for laboratory rodents: final report of the American Institute of Nutrition ad hoc writing committee on the reformulation of the AIN-76A rodent diet. *J Nutr*, *123*(11), 1939-1951. <https://doi.org/10.1093/jn/123.11.1939>
